# Wide Range of Brachyceran Fly Taxa Attracted to Synthetic and Semi-Synthetic Generic Noctuid Lures and the Description of New Attractants for Sciomyzidae and Heleomyzidae Families

**DOI:** 10.3390/insects14080705

**Published:** 2023-08-14

**Authors:** Antal Nagy, Patrik Katona, Attila Molnár, Zoltán Rádai, Miklós Tóth, Kálmán Szanyi, Szabolcs Szanyi

**Affiliations:** 1 Institute of Plant Protection, Faculty of Agricultural and Food Sciences and Environmental Management, University of Debrecen, Böszörményi út 138, H-4032 Debrecen, Hungary; nagyanti@agr.unideb.hu (A.N.); szanyi.szabolcs@agr.unideb.hu (S.S.); 2 Independent Researcher, Hold utca 1, 2220 Vecsés, Hungary; 3 Department of Zoology and Ecology, Hungarian University of Agriculture and Life Sciences, Péter Károly utca 1, H-2011 Gödöllő, Hungary; 4 Department of Dermatology, University Hospital Düsseldorf, Medical Faculty, Heinrich-Heine-University, 40225 Düsseldorf, Germany; 5 Institute of Metagenomics, University of Debrecen, Nagyerdei körút 98, H-4032 Debrecen, Hungary; 6 Plant Protection Institute, CAR, ELKH, Herman Otto u. 15, H-1022 Budapest, Hungary; 7 Department of Hydrobiology, Faculty of Science and Technology, University of Debrecen, Egyetem tér 1, H-4032 Debrecen, Hungary; 8 Juhász-Nagy Pál Doctoral School of Biology and Environmental Sciences, University of Debrecen, Egyetem tér 1, H-4032 Debrecen, Hungary

**Keywords:** phenylacetaldehyde, isoamyl alcohol, Diptera, sampling, allelochemicals, generic lures

## Abstract

**Simple Summary:**

Plant volatile traps designed for Lepidoptera pests caught a large number of flies as non-target insects that provided an opportunity to study their odour preferences. The tested isoamyl alcohol-based semisynthetic bisexual lure containing red wine as an organic component attracted flies of 10 families, including economically important ones such as Drosophilidae and Ulididae, and it is the first reported attractant of the Sciomyzidae family. Although our phenylacetaldehyde-based synthetic lure attracted less families with lower abundance, it was efficient against the Empididae and Milichiidae families. In the case of Heleomyzidae flies, both lures were efficient, and these are the first published attractants of this family. These new data on the chemical ecology of flies serve as a basis for further studies and may be utilized in the development of sampling methods used in biological and integrated pest management, and faunistic and ecological studies.

**Abstract:**

During field tests implemented in Transcarpathia (West Ukraine) in 2015, 6501 specimens belonging to 26 Brachyceran fly families were collected with traps baited with generic lures (originally developed for noctuid moths) based on fermenting liquid and floral compounds. Isoamyl alcohol-based baits generally attracted more flies than phenylacetaldehyde-based baits and unbaited controls; however, the phenylacetaldehyde-based traps were the most attractive to the Empididae and Milichiidae families. The isoamyl alcohol-based semisynthetic lure showed significant attractivity to the families of Muscidae, Ulidiidae, Sarcophagidae, Calliphoridae, Sciomyzidae, Heleomyzidae, Drosophilidae, Phoridae and Platystomatidae. Additionally, isoamyl alcohol-based semisynthetic lure is the first reported attractant of the Sciomyzidae family. Since our phenylacetaldehyde-based floral lure was also attractive to Heleomyzidae flies, both types of lures can be seen as the first known attractants of this family.

## 1. Introduction

Diptera is one of the most species-rich insect taxa playing an important role in natural ecosystems as well as in human life [1]. On the one hand, there are several beneficial predatory, parasitic, decomposer and pollinator species among them, while on other, flies have outstanding importance in veterinary and human health by transmitting devastating diseases, and causing significant economic loss in forestry and agriculture, both in the field and stores [2]. Monitoring the presence and population dynamics of different fly pest species is an important part of IPM (integrated pest management). Different fly pests may be trapped with chromotropic-, pheromone-, and other attractant-baited traps in monitoring, and management strategies, such as mass-trapping, male-annihilation and lure-and-kill methods [3,4,5,6,7].

In Europe, flies cause significant damage to crops, vegetables, and fruits. The economically most important groups are the multivoltine Chloropidae and Agromyzidae species, developing in cereals and maize; Anthomyiidae species, living in vegetables (onion, cabbage) and rape (e.g., Delia radicum L., Delia floralis Fallén); fruit flies (Tephritidae: Rhagoletis spp.) damaging cherries, sour cherries and walnuts; Ceratitis capitata Wied. feeding on various fruits from orange to peach [8]; and the invasive Drosophila suzukii Mat. (Drosophilidae) endangering various fruits [9,10].

In the Pherobase [11], data on 29 Brachycera and 9 Nematocera families can be found. Pheromones, colour preference and attractants of many Dipteran pests are known. Yellow sticky traps are useful tools for monitoring fruit flies (e.g., Rhagoletis spp.) and shore flies (Ephydridae: Scatella stagnalis Fallén) [12], while house flies (Muscidae) prefer the white-coloured traps. Baits can increase the efficiency of colour traps, like in the case of Delia flies [13], Rhagoletis species [14] and house flies [15]. Flies can be attracted to various compounds, from primer alcohols to amines, and even sibling taxa may have highly different preferences due to their different life forms, habitat preferences and coevolution with different hosts. For example, two Anthomyiidae genera, Delia and Anthomyia, show different preferences: Delia species could be trapped mainly with 2-phenylethanol and valeric acid [16], while Anthomyia species could be attracted by cantharidin and secondary alcohols [17,18,19,20].

To develop traps with generic lures to catch noctuid moths and other dangerous Lepidopteran pests (e.g., European corn borer Ostrinia nubilalis Hübn.), the attractivity of many compounds and mixtures was previously tested by us. In these studies, isoamyl alcohol-based and phenylacetaldehyde-based lures showed the highest efficiency [21,22,23,24,25,26,27,28,29].

Besides lepidopteran pest and non-pest species summarized in [30], and [21], a large amount of non-target Hymenoptera, Neuroptera, Coleoptera and even Diptera specimens were captured during intensive field studies. In the present paper, our goal is to report on the Diptera samples collected in Velyka Dobron’ (Transcarpathia, West Ukraine) in 2015, and evaluate the effect of the most efficient lures on different brachyceran families.

## 2. Materials and Methods

### 2.1. Study Area

The samplings were carried out in Velyka Dobron’, belonging to the Transcarpathian region of West Ukraine. The study area was a patchy agricultural landscape of the Bereg Lowland, consisting of a mosaic of natural and semi-natural patches, which preserve the remains of marshy, boggy habitats, such as an oak–ash–elm hardwood gallery forest (Fraxino-pannonicae-Ulmentum) dominated by Quercus robur L., Fraxinus angustifolia subsp. Pannonica Soó et Simon, Ulmus laevis Pall. and Populus canescens (Aiton) Sm. species, oak–hornbeam forest, and numerous xerophilous silver lime–oak forests. Forest fringes, mesic and humid forest clearings, willow scrubs and even agricultural habitats, such as hedges, country roads, roadsides, channels, arable lands, and stubble fields also increase the habitat diversity of the sampling area [30]. Samplings were made along a linear transect in the forest edge of a hardwood gallery forest north of the Velyka Dobron’ village.

### 2.2. Baits and Sampling

We used the standard CSALOMON^®^ VARL+ funnel traps, produced by the Plant Protection Institute, CAR ELKH (Budapest, Hungary) (www.csalomontraps.com accessed on 1 August 2023) [31,32]. The two types of generic noctuid lures tested were the same as described earlier [28,30] and were produced at the same institute where the traps were made: a semisynthetic bisexual lure (SBL) containing isoamyl alcohol (3-methyl-1-butanol; frequently occurring in fermenting molasses) + acetic acid + red wine (1:1:1), and a synthetic floral lure (FLO) containing synthetic floral compounds (see below). Traps without baits were also set out for control. All compounds used were bought from Sigma-Aldrich Ltd. (Budapest). The purity of phenylacetaldehyde was ≥95%, while in case of the other compounds, it was between 98 and 99%. Red wine was produced by G. Veres in Szekszárd from different grape sorts: Bluefrankish (70%), Merlot (15%), Kadarka (7.5%) and Blauburger (7.5%), and its alcohol content was 13.6–13.8%, with a volatile acid (acetic acid) content of 0.4–0.6 g/L.

Polypropylene tubes with a 4 mL capacity were used as dispensers for the SBL [33]. The synthetic compounds were administered on the dental rolls inside the tubes. The upper, larger opening of the tube was closed with its lid. The bait mixture could evaporate through the lower smaller opening of 4 mm diameter, which was opened after being set out.

The FLO traps were baited with two separate polyethylene bag dispensers [32]. One of them contained a mixture (1:1:1, 0.6 mL) of phenylacetaldehyde, eugenol and benzyl acetate [24], while the other contained a mixture (1:1, 0.4 mL) of phenylacetaldehyde and (E)-anethol [25].

The sampling was carried out between the 17th of May and the 1st of November 2015 (24 weeks). Traps were checked and emptied once a week, and trapped insects were killed by an insecticide strip (Vaportape^®^ II). Each bait type was used in four repetitions, thus 12 (4 × 3) traps were placed in the survey area, at a 20 m distance from each other, and hung at ca. 1.8–2 m high. Traps were rotated at the time of checking, to eliminate the bias caused by the trap’s location. The baits were fixed under the top of the trap and were replaced after four weeks. The collected materials were deep-frozen (−20 °C) and stored until the processing. 

The collected specimens were identified at family level, using Lomo MBS-10 and Leica MZ12.5 stereoscopic microscopes, based on the keys provided by [1,34]. The ratio of unidentifiable specimens was 0.6%, which were excluded from further analyses. For taxonomy and taxon names, the work by Pape at al. [35] was followed. The collected material was placed at the Hungarian Natural History Museum collection.

### 2.3. Statistical Analyses

The number of individuals caught was determined for each sample of the different trap types (SBL, FLO and Control). The efficiency of the lures was compared based on the mean catches per trap. A total of 14 of 26 sampled families were represented with more than 30 individuals/trap in total (Muscidae, Ulidiidae, Sarcophagidae, Calliphoridae, Empididae, Anthomyiidae, Sciomyzidae, Milichiidae, Chloropidae, Heleomyzidae, Drosophilidae, Phoridae, Lauxaniidae, and Platystomatidae). The data sets studied did not fulfil the assumptions of the parametric tests, checked with the Levene-test (homogeneity of variances) and Q-Q plots (normality); thus, the non-parametric Kruskal–Wallis test was used. When the latter showed significant differences, the Mann–Whitney U-test was used for paired comparisons of the treatments. Calculations were carried out with SPSS 21.0 software package (SPSS for Windows 2001; [36,37]). The temporal changes in the number of individuals caught were described with graphs.

## 3. Results

Although most flies were attracted to the SBL, the unbaited control traps also caught more than 300 individuals. During the study, 6539 flies were caught, and the number of specimens that could be identified at the family level was 6501. These flies belonged to 26 families. The most abundant families, represented by more than 500 individuals, were Muscidae, Ulididae, Sarcophagidae and Calliphoridae, respectively. Contrarily, only single individuals of the Stratiomyidae, Tabanidae, Hybotidae and Scathophagidae families were caught (Table 1).

**Figure 1:**
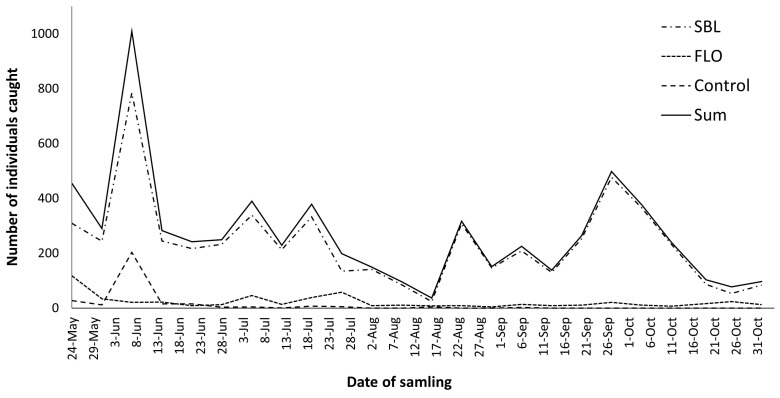
Temporal changes in total number of individuals caught by traps baited with different lures (SBL, FLO, unbaited control) and the total in 2015.

Temporal changes in the number of individuals caught showed two peaks in early June and late September, while in mid-August, the abundance of flies decreased radically. The SBLs attracted flies in both periods of their phenology (before and after mid-August), while FLO traps were efficient mainly from May to July. Unbaited control traps caught individuals also in the first phenological period, when flies were especially abundant in SBL traps (Figure 1).

**Figure A1:**
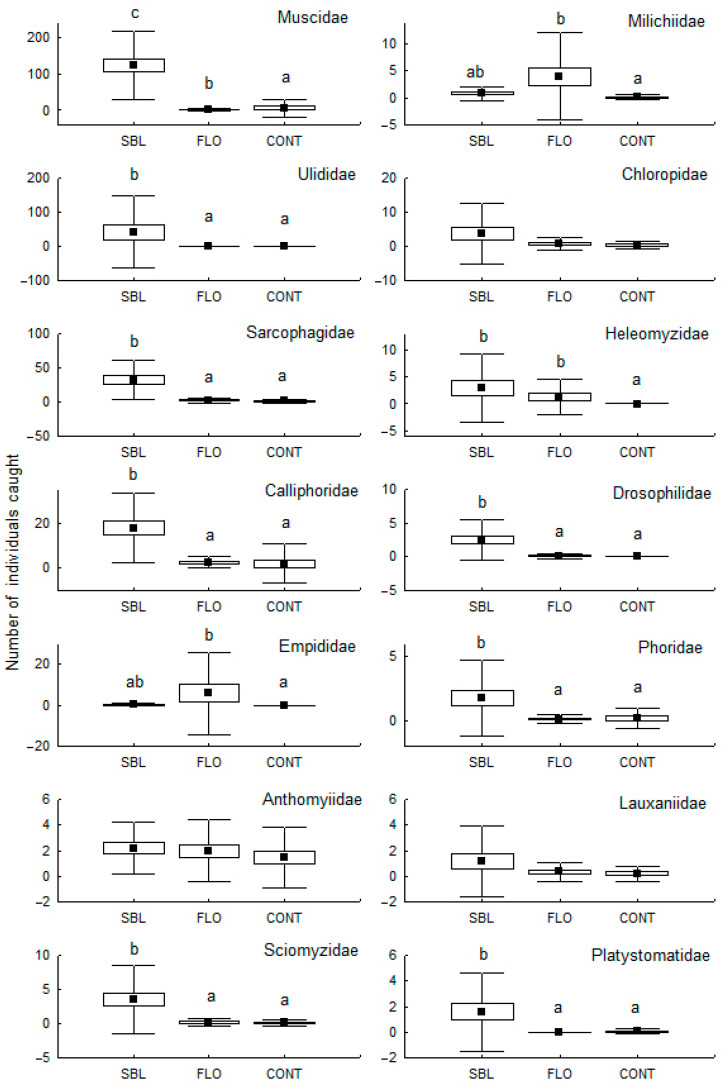
Mean number of individuals (black box with ±SE: empty box; and ±SD: whisker) belonging to families of Brachycera caught by different traps with numbers larger than 30. Different small letters refer to significant differences based on the Mann–Whitney U-test (*p* < 0.05).

The SBL traps attracted significantly more specimens (Appendix A Figure A1) belonging to the family of Muscidae, Ulididae, Sarcophagidae Calliphoridae, Sciomyzidae, Drosohilidae, Phoridae and Platystomatidae than both the FLO and the unbaited control traps. FLO traps were efficient against Empididae and Milichiidae, while flies belonging to Heleomyzidae family were trapped with both lures tested. Considering Anthomyiidae, Chloropidae and Lauxaniidae, significant differences could not be detected in the attractivity of the lures (Appendix A Figure A1).

The specimens of the less-abundant families of Syrphidae and Tachinidae were caught mainly with FLO traps, while SBL traps were more attractive to Microphoridae species. Specimens of Odiniidae, Periscelididae, Pallopteridae, Tabanidae, Hybotidae and Scathophagidae appeared only in SBL traps. One fly belonging to the family of Statiomyidae was caught only with an FLO trap; single Dolichoporidae specimens were found in a FLO and an unbaited trap, while representatives of the Piophilidae family appeared only in unbaited traps (Table 1).

## 4. Discussion

General lures designed to attract noctuid moths also attracted a high number of brachyceran flies belonging to 26 families in the present study. Between the two lures considered, the semisynthetic bisexual lure (SBL) containing isoamyl alcohol, acetic acid and red wine (1:1:1) was more efficient. The attractivity of this ternary mixture to moths was already known in a wide range of Geometridae, Thiatiridae and Erebidae species and noctuid moths, mainly belonging to the Noctuinae, Xyleninae and Hadeninae subfamilies [21,26,29,38]. Here, its attractivity to nine dipteran Brachycera families was proven and it is reported here for the first time in the families of Ulidiidae, Sarcophagidae, Calliphoridae, Sciomyzidae, Heleomyzidae and Platystomatidae. In addition, to our knowledge, this ternary lure is the first reported attractant in Heleomyzidae and Sciomyzidae families. In the Ulidiidae family, only the efficiency of methyl eugenol and cuelure against *Euxesta annonae* Fabr. have formerly been studied in Hawaii [39,40]. However, since some other species of the family have significant economic importance, such as *Euxesta stigmatias* Loew, whose larvae attack maize in tropical and subtropical America [41], and *Tetanops myapaeformis* Ord. (sugar beet root maggot), which is a serious pest of sugar beet in North America [42], it would be worthwhile to make further studies with the compounds of the tested ternary mixture (SBL) on these pests. Since the composition of the SBL is similar to that of fermenting liquids, its attractivity showed that these fly families prefer damaged parts or leaking sap of different plants. In other cases, this kind of volatile preference is characteristic for species living in forests and forest edges [21,26,28,43].

Specimens belonging to nine other families (Syrphidae, Tachinidae, Microphoridae, Odiniidae, Periscelididae, Pallopteridae, Tabanidae, Hybotidae, and Scathophagidae) were also found in SBL traps, but with low abundances.

Attractiveness of isoamyl alcohol alone was already known in the Phoridae (e.g., *Megaselia* sp.) [44] and Syrphidae (e.g., *Eupeodes volucris* Ost.-Sack.) [45] families. Beyond that, its attractivity to the family of Scatopsidae (*Swammerdamella brevicornis* Mei.), Drosophilidae (*Drosophila melanogaster* Meig.) [44] and Tephritidae as a component of multicomponent lures is also known. Isoamyl alcohol also attracted *Rhagoletis zephyria* Snow and *Anastrepha suspensa* Loew [46,47]; it is a kairomone of *C. capitata* and *Rhagoletis pomonella* Wal. [48,49,50], and a pheromone component of *Bactrocera umbrosa* Fabr. [51].

In total, the attractivity of acetic acid is known in the case of 16 species of 8 Brachycera families, while a synanthropic parasitic eye gnat (*Hippelates collusor* Tow., Chloropidae) with veterinary importance is attracted to a mixture containing acetic acid [52]. Both *D. melanogaster* and *D. suzukii* (Drosophilidae) are reported to be attracted to lures containing acetic acid [44,53,54,55,56]. This is also reported in other important families involving many dangerous pests and vectors as Muscidae (*Morella* sp. [57]) and Tephritidae (*Anastrepha ludens* Loew [58]; *C. capitata* [57]). Moreover, its combination with isobutanol (2-methyl-1-propanol) attracted yellowjacket species (Hymenoptera: Vespidae) [59,60,61,62,63,64,65]. 

In Lepidoptera, the combination of isoamyl alcohol with acetic acid has been found to attract both sexes of several noctuid pests in North America and Europe [66,67,68,69,70]. The other lures tested, which contained compounds of flower scents, attracted fewer families with lower densities. The synthetic lures tested were effective for the collection of specimens of the Empididae, Heleomyzidae and Milichiidae families. 

In the case of Heleomyzidae, the attractivity of FLO lure is also reported here for the first time but there were no significant difference between the two tested lures. In the Empididae family, only the attractivity of methyl salicylate to *Rhamphomyia gibba* Fall. [71] and cuelure to *Hemerodromia stellaris* Mel. [39] are known. Considering Milichiidae, only the effect of a mixture of geraniol, 2-heptanone, 2-nonanol, and (E)-2-octen-1-yl acetate was proven on *Desmometopa* species, which are well-known flies feeding on honeybees caught by spiders. Additionally these flies are the main pollinators of the *Ceropegia sandersonii* Dec. (Apocynaceae) plant, which lures kleptoparasitic flies into their biological fly-pollinated pitfall-trap flowers by simulating the scent of an injured honeybee [72]. We found no previous reports on the attractivity of the floral compounds of the FLO lure considered in the present study.

As for Lepidoptera phenylacetaldehyde, it is a well-known floral compound that attracts many moths, such as the families of Crambidae, Noctuidae and Sesiidae [70,73,74,75,76,77,78]. Also, it was shown that phenylacetaldehyde is a chemical attractant for common green lacewings *Chrysoperla carnea* Step. s.l. (Neuroptera: Chrysopidae) [79]. Contrarily, the compound’s attractivity to dipterans has been revealed only in three Nematocera species, belonging to the Bibionidae (*Plecia nearctica* Har.: [80]), Culicidae (*Culex pipiens* Lin.: [81]) and Scatopsidae (*Coboldia fuscipes* Meig.: [82]) families.

The effects of the other compounds of the FLO lure are mainly unknown on brachyceran species. Only the attractivity of eugenol to *Musca domestica* Lin. (Muscidae [83]) and benzyl acetate to *Daucus ciliatus* Loew (Tephritidae [84]) has been already reported.

In conclusion, the effectiveness of the tested lures and their components is poorly known against Brachyceran taxa, especially in the temperate zone. In the present study, two tested lures attracted species of 11 brachyceran families, containing species with importance in plant protection and for veterinary. Further studies with these lures may serve as a basis for the monitoring and management of fly pests, vectors and parasites.

## Figures and Tables

**Table 1:** Number of brachyceran individuals caught with traps baited with different lures and control traps by fly families. Families with N < 30 were excluded from comparisons of the tested lures and separated with a line.

Family	Total	CONTROL	SBL	FLO
Total number of individuals	6501	306	5656	543
Muscidae	3137	147	2946	44
Ulidiidae	1001	9	990	2
Sarcophagidae	873	35	780	58
Calliphoridae	536	48	425	63
Empididae	145	0	4	141
Anthomyiidae	136	35	53	48
Sciomyzidae	118	3	85	5
Milichiidae	115	3	19	96
Chloropidae	100	10	89	16
Heleomyzidae	93	0	70	30
Drosophilidae	63	0	60	3
Phoridae	49	4	42	3
Lauxaniidae	41	5	28	8
Platystomatidae	39	1	38	0
Syrphidae	18	0	4	14
Tachinidae	13	0	4	9
Microphoridae	7	2	4	1
Odinidae	4	0	4	0
Piophilidae	3	3	0	0
Dolichopodidae	2	1	0	1
Periscelididae	2	0	2	0
Pallopteridae	2	0	2	0
Statiomyidae	1	0	0	1
Tabanidae	1	0	1	0
Hybotidae	1	0	1	0
Scathophagidae	1	0	1	0

## Data Availability

Raw data supporting the results in the paper were uploaded into the Zenodo open repository: Antal Nagy, Patrik Katona, Attila Molnár, Zoltán Rádai, Miklós Tóth, Kálmán Szanyi, and Szabolcs Szanyi (2023). Wide range of Brachyceran fly taxa attracted to synthetic and semi-synthetic generic noctuid lures and the description of new attractants for Sciomyzidae and Heleomyzidae families—RAW Data [Data set]. Zenodo. https://doi.org/10.5281/zenodo.7938981 (accessed on 1 August 2023).
